# Ocular disorders as the prevailing manifestations of antiphospholipid syndrome: a case series

**DOI:** 10.1186/1757-1626-2-159

**Published:** 2009-10-20

**Authors:** Evangelia Tsironi, Nikolaos Gatselis, Maria G Kotoula, Kalliopi Zachou, Maria Pefkianaki, Fani Zacharaki, Dimitrios Z Chatzoulis, George N Dalekos

**Affiliations:** 1 Department of Ophthalmology, Medical School, University of Thessaly, Larissa, 41110, Greece; 2 Department of Medicine and Research Lab of Internal Medicine, Medical School, University of Thessaly, Room No 23, 41110 Larissa, Greece; 3 Research Group of Investigational Medicine, Institute of Biomedical Research and Technology, Centre for Research and Technology-Thessaly (CE.RE.TE.TH), Larissa, 41110, Greece

## Abstract

**Introduction:**

Antiphospholipid syndrome is an autoimmune disorder characterized by either a history of vascular thrombosis (one or more clinical episodes of arterial, venous, or small vessel thrombosis in any tissue or organ) or pregnancy morbidity in association with the presence of antiphospholipid antibodies. The systemic features of the syndrome are characterized by large variability depending on the affected organ(s). Among them, neurological and behavioural disturbances, dermatological features as livedo reticularis and renal, ocular, liver or valvular heart manifestations have been reported in antiphospholipid syndrome patients. However, studies on the frequency and clinical presentation of the ocular manifestations as the prevailing (first) sign of antiphospholipid syndrome in patients suffering from "unexplained" ocular disease are missing. Herein, we present three cases suffering from unexplained ocular disease as first manifestation of antiphospholipid syndrome.

**Case presentation:**

All the three patients were referred to our department because of unexplained ocular features from the anterior or posterior segment and unexplained neuro-ophthalmologic symptoms. The first patient had bilateral retinal occlusive disease, the second and the third patient had unilateral nonarteritic anterior ischemic optic neuropathy with macular oedema. Moderate to high levels of antiphospholipid antibodies were detected in all of them at baseline as well as 6 to 12 weeks after initial testing confirming the presence of antiphospholipid antibodies. Anticoagulant treatment with acenocoumarol was instituted resulting in stabilization and/or improvement of ocular signs in all of them.

**Conclusion:**

Due to the important diagnostic and therapeutic implications of antiphospholipid syndrome, the possibility of ocular features as the first clinical manifestation of antiphospholipid syndrome should be kept in mind of the physicians particularly in patients with no evident risk factors for ocular disease. In this case, prompt anticoagulant treatment and close follow-up seem to be essential for vision salvation and stabilization.

## Introduction

The antiphospholipid syndrome (APS) is an autoimmune disorder characterized by either a history of vascular thrombosis (one or more clinical episodes of arterial, venous, or small vessel thrombosis in any tissue or organ) or pregnancy morbidity in association with the presence of antiphospholipid (aPL) antibodies [1-3]. These antibodies namely, anticardiolipin (aCl) antibodies, lupus anticoagulant (LA), or antibodies against beta2-glycoprotein I (anti-b2GPI) either of IgG or IgM isotype have been recently established as the laboratory criteria for the diagnosis of definite APS [1].

The systemic features of the syndrome are characterized by large variability depending on the affected organ(s). In this context, neurological and behavioral disturbances, dermatological features as livedo reticularis and renal, ocular, liver or valvular heart manifestations have been reported in APS patients [1,3,4].

Ocular involvement in APS includes a broad spectrum of manifestations from the anterior and posterior segment or the presence of neuro-ophthalmologic features [4-9]. In brief, conjuctival telangiectasia or conjuctival microaneurysms, episcleritis, limbal or filamentary keratitis and iritis have been described as the APS ocular features from the anterior segment [8,9], vitritis, retinal detachment, posterior scleritis, branch or central retinal vein occlusion, bilateral choroidal infarction, cilioretinal artery occlusion, venous tortuosity, retinal hemorrhages, cotton-wool spots and central serous type chorioretinopathy from the posterior segment [4,8] and monocular or bilateral transient visual loss, transient visual field loss, ischemic optic neuropathy and progressive optic nerve atrophy as the neuro-ophthalmologic features of APS [4,5].

However, studies on the frequency and clinical presentation of the ocular manifestations as the prevailing (first) sign of APS in patients suffering from "unexplained" ocular disease are missing. Recently, we have reported a patient with "unexplained" bilateral choroidal embolization as the first clinical manifestation of the underlying APS [10]. Herein, we report 3 additional cases suffering from "unexplained" ocular disease as first manifestation of APS in a well-defined geographical region of Greece [11,12] and describe the clinical manifestations, diagnosis and course, imaging findings, management and the final outcome of patients as similar data is missing.

## Case presentations

In all 3 cases the ocular features considered "unexplained" after an extensive investigation which included the following: absence of systemic arterial hypertension, diabetes mellitus and/or impaired glucose tolerance, hyperlipidemia, heart disease including embolizating cardiomyopathy, carotid atherosclerosis, multiple sclerosis and obvious infections. Therefore, the patients were referred to the department of medicine for thorough investigation. Special attention was paid to the presence of past or present history of arterial hypertension (systolic blood pressure greater than 135 mmHg and diastolic blood pressure greater than 85 mmHg on several occasions or taking antihypertensive drugs), diabetes mellitus (considered present if fasting serum glucose levels were equal or above 126 mg/dl in more than one occasion or random serum glucose levels equal or above 200 mg/dl in more than one occasion), impaired glucose tolerance (considered present if repeatedly fasting serum glucose levels were between 110 and 125 mg/dl or serum glucose levels after 2 hours of 75 gr glucose ingestion in between 126 and 199 mg/dl), hyperlipidemia defined as fasting serum triglycerides equal or greater than 200 mg/dl or fasting serum cholesterol between 200 and 240 mg/dl accompanied by increased LDL cholesterol levels (above 160 mg/dl) or cholesterol levels greater than 240 mg/dl irrespective of LDL cholesterol levels [13]. Heart disease was investigated for the presence of clinical, electrocardiographic and echocardiographic signs of valvular disease, myocardial infarction, atrial fibrillation, ventricular arrhythmias, conduction defect blocks, and left atrial enlargement. Significant atherosclerosis was considered when carotid echo-Doppler investigation revealed flow reduction greater than 60%. Absence of multiple sclerosis was defined if there was no clinical evidence of the disease and magnetic resonance imaging of brain and spinal cord were normal. The absence of infections was defined by thorough clinical investigation, repeatedly negative blood and urine cultures and seronegativity by specific enzyme linked immunosorbent assays (ELISAs) and molecular techniques for several bacteria and viral antigens including human immunodeficiency virus (HIV), herpes simplex virus (HSV), hepatitis B virus (HBV) and hepatitis C virus (HCV) [14,15].

### Case report 1

**Figure 1:**
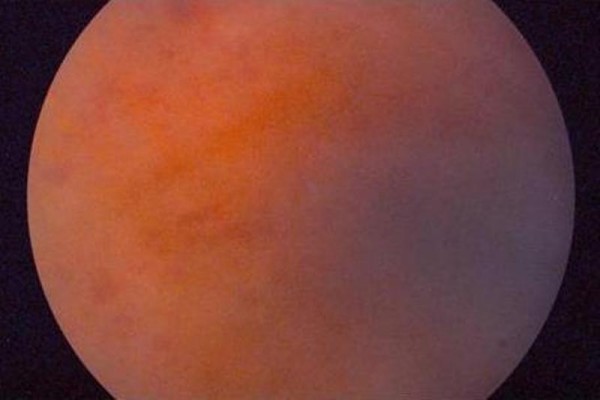
**RE fundus photography shows vascular occlusion and vitritis *(Case 1)***.

**Figure 2:**
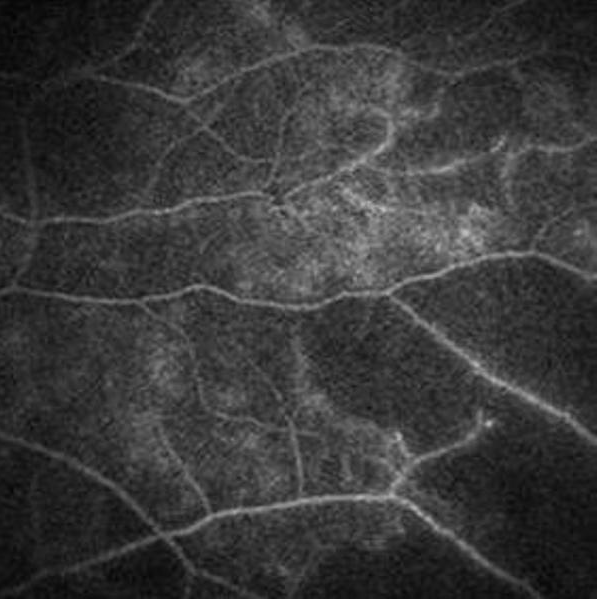
**RE fluorescein angiography shows peripheral ischemic vasculitis (*Case 1)***.

A 39-years-old Greek male patient of Caucasian origin referred to our department due to floaters perception bilaterally since 3 weeks. His past history was unremarkable. His visual acuity was 20/20 bilaterally. Anterior chamber of the right eye (RE) presented with a mild flare while anterior chamber of the left eye (LE) appeared normal. Fundus examination revealed moderate vitritis in both eyes and extensive arterial occlusive vasculitis and retinal haemorrhages in the mid-periphery (Figure 1), findings that were confirmed by fluorescein angiography (Figure 2).

Physical examination, and routine haematological and biochemical laboratory work-up including erythrocyte sedimentation rate were unrevealing. Repeated blood cultures proved negative for bacterial and fungal infections, while serology tests for several bacteria, viruses and toxoplasma tested negative. Aqueous humor, taken by RE paracentesis, tested also negative by polymerase chain reaction for HSV1/2, CMV, V-ZV, EBV, mycobacterium tuberculosis and bacteria infections. Abdominal ultrasonography, computed tomographies scan of the lungs, the upper and lower abdomen as well as the retro-peritoneal space, magnetic resonance imaging of the brain and carotid Doppler-testing revealed no findings revealed no findings. Concentrations of C3 and C4 components of the complement, angiotensin converting enzyme, immunoglobulins, rheumatoid factors and C-reactive protein and 24 hours urine calcium concentrations were within normal limits, while antinuclear (ANA), smooth muscle (SMA), anti-mitochondrial (AMA), anti-dsDNA and anti-neutrophil cytoplasmic antibodies (ANCA)were undetectable.

Further testing gave a high positive result (231 BI; cut-off: 100 BI) for IgG-aCL antibodies by using a highly sensitive, specific and internationally accepted quantitative solid phase ELISA as we described previously [2,10,16-18], while LA ratio determined using the diluted Russell's viper venom test according to the manufacturer's instructions (HemosIL™, Instrumentation Laboratory Company-Lexington, USA) was 1.6 (moderate titre; normal values < 1.2) [10]. IgG and IgM anti-b2GPI antibodies were determined by a commercial ELISA according to the manufacturer's instructions (QUANTA Lite™ β_2_-GPI IgG and IgM, INOVA Diagnostics; cut-off: 20 SU) and were negative. His prior history of arteriovenous thrombotic events (myocardial infraction, ischemic stroke, pulmonary emboli, deep venous thrombosis of legs, arms or internal organs) or neuropsychiatric disorders (convulsions, migraine, memory loss, chorea or psychosis) was not contributory for previous APS symptoms. Thorough investigation for clinical and laboratory criteria of autoimmune rheumatic diseases as systemic lupus erythematosus, Sjogren's syndrome, rheumatoid arthritis, systemic angiitis, sarcoidosis and seronegative arthritis including Behcet's syndrome and adult Still's disease was unrevealing. The patient started promptly anticoagulant treatment with acenocoumarol with a target INR of 2.5-3.0. The IgG-aCL titre remained high positive 6-12 weeks after the first test (258 BI), whereas LA test showed again moderate titre (1.65) confirming the presence of APS. At a 5-month follow-up the patient underwent pan-retinal laser photocoagulation since bilateral retinal neovascularization was found. At the 20th month of follow-up he is still in anticoagulant therapy with very good treatment-efficacy regarding the INR values and without side effects; his visual acuity remains 20/20, without vitritis and no sites of neovascularization in fluoroscein angiography.

### Case report 2

**Figure 3:**
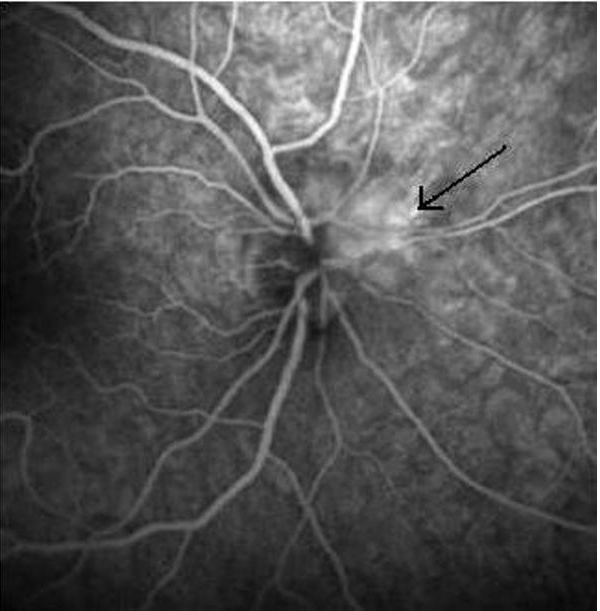
**RE fluorescein angiography in *Case 2 *shows optic disk segmental hyperfluorescence (arrow) in late phases (arrow)**.

A 32-years-old Greek female of Caucasian origin was admitted due to RE floaters perception and visual obscuration. Her past medical history was not contributory. She was not smoker or alcohol consumer. Her visual acuity was 20/25 in the RE and 20/20 in the LE. The anterior segment and intraocular pressure tested normal. Fundus examination revealed a partial nasal edema of the RE optic disc confirmed by fluoroscein angiography (Figure 3). No pathological findings were detected from the LE fundus examination. Visual fields (30-2, Humphrey) revealed normal responses in the LE and an inferior sectional temporal scotoma in the RE. Based on the above findings the diagnosis of a mild nonarteritic anterior ischemic optic neuropathy (NAION) was made.

Physical examination and all thorough investigation as it was reported in *case *1 were unrevealing. Further testing however, gave a high positive result for IgG-aCL antibodies, (207 BI), while LA tested negative and IgG anti-b2GPI antibodies positive (35 SU). The questionnaire for signs or symptoms suspicious of prior APS history was negative. The patient started anticoagulant treatment with acenocoumarol. The IgG-aCL titres remained high positive (230 BI and 277 BI) 6 and 12 weeks after first testing. At the 2-month follow-up the RE optic disc oedema resolved leaving nasal partial optic disc atrophy and the visual field revealed partial improvement of the defect. The patient was evaluated at 6-month intervals and in the last follow-up -2 years later- her visual acuity remains stable. She is still under anticoagulant treatment with excellent treatment-efficacy regarding the INR values and without side effects.

### Case report 3

A 62-years-old Greek male patient of Caucasian origin was referred to our department for LE reduced vision due to NAION, diagnosed by his private ophthalmologist. His best-corrected visual acuity was 20/20 (RE) and 20/400 (LE). Visual fields revealed normal responses in RE and a lower altitudinal defect in LE. Fluorescein angiography showed a lower segmental hyperfluorescence of the LE optic disk in the late phases. He was not smoker or alcohol consumer. As in the previous two cases, physical examination and all investigations were unrevealing. Further testing however, gave a moderate positive moderate result for IgG-aCL antibodies (178 BI), which became strong positive at 6 weeks determination after the first test (216 BI). LA and anti-b2GPI antibodies tested negative. The patient started anticoagulant treatment with acenocoumarol. Follow-up at 1-month intervals showed a slow progressive improvement of the LE visual acuity reached the 20/64 level within 5 months period, which is so far the last follow-up of the patient. His LE optic disk was found pale.

## Discussion

These cases demonstrate that the ocular features as the first presenting sign of APS though rare, should alert the clinicians for APS possibility, particularly when conventional risk factors have been excluded and the onset of ocular symptoms are insidious [5,7,8,19]. Although the involvement of anterior segment is not rare, the manifestations from the posterior segment such as vasculitis, vitritis, retinal detachment, posterior scleritis and central retinal artery occlusion appear to be more prominent among APS patients [4,8]. Of these, the most common and the most serious is vasculitis, which can result in vaso-occlusive disseminated retinopathy as in our first case. Of note, APS-related retinal artery occlusions are characterized by a strong tendency to retinal neovascularization, as in our first case [9]. In these cases, pigment epithelial window defect has been reported as the most frequent finding in fluorescein angiography. Neuro-ophthalmological manifestations related to APS are also common as monocular or bilateral transient visual loss, transient visual field loss or anterior ischemic optic neuropathy [4,6,8]. In our case series, 2 out of 3 patients presented with NAION, one female young adult (case 2) and one healthy middle aged male (case 3).

Previous studies on aPL prevalence in ocular vascular events are scarce, mostly retrospective having been performed in old patients with concurrent risk factors. In a recent study, Cobo-Soriano et al [7] found aCL positivity in 9/40 (22.5%) patients with retinal thrombosis without main conventional risk factor for thrombosis though, aCL titres were that of low positive in most instances and APS was confirmed finally in 3/9 aCL positive patients [7]. Furthermore, in the latter study no detailed data is given in order to assess whether these manifestations were the first features of APS [7]. In another recent pilot study, Marcucci et al [19] reported a prevalence of 9.7% for LA and 14.6% for aCL in a group of 41 unselected patients with retinal artery occlusion. However, aPL prevalence in that study was probably overestimated as it was based on a single determination, while the statistically significant association of these antibodies with the presence of retinal artery occlusion was not maintained after multivariate analysis [19]. From the few studies reported in the literature, only hyperhomocysteinemia among thrombophilic factors, seems to be an independent risk factor for retinal artery occlusion [19,20], whereas data available on other thrombophilic factors including aPL antibodies are still inconclusive. However, the aim of our study was different from the abovementioned studies as we tried for the first time to report cases suffering from "unexplained" ocular disease as the first manifestation of APS.

From the clinical point of view we believe that the detection of aPL antibodies could be of interest and relevance in medicine as it can provide -at least in part- a new overview of the pathogenesis of some ocular diseases particularly in young patients without known evident risk factors. In addition, although the last revised criteria for the classification of APS [1] do not include several well-known features which are associated with APS, we believe that the repetitive detection of aPL antibodies (6 and/or 12 weeks from first testing) has important diagnostic and therapeutic implications as seems to confirm APS diagnosis and subsequently indicates the need of anticoagulation therapy, which can prevent not only future events in the eyes but also episodes in other vital organs [1,2,4,5,10]. APS prognosis clearly depends on the severity of the disease, the thrombotic events, recurrences and the side effects of treatment [3]. However, the prognosis of ocular diseases due to APS is not well documented, as long-lasting prospective studies are missing. Though there is still no definite agreement for the duration of anticoagulant treatment, it seems rational and prudent to treat these patients lifelong targeting an INR between 2.5 and 3.0, while the role, if any, of corticosteroids is unclear.

## Conclusion

As APS diagnosis has important diagnostic and therapeutic implications, we do believe that the possibility of APS should be considered by the internists in cases with "unexplained" ocular manifestations who have no evident risk factors since prompt administration of anticoagulant treatment is known to be essential for APS outcome and potentially for vision salvation and stabilization.

## Consent

Written informed consents were obtained from the patients for publication of these case reports and accompanying images. Copy of the written consents are available for review by the Editor-in-Chief of this journal.

## Competing interests

The authors declare that they have no competing interests.

## Authors' contributions

GND, EZ and DZC had the original idea for the study, wrote the study protocol, and along with NG wrote the paper. KZ and NG did the laboratory work and investigate the patients from the clinical point of view in the department of medicine. ET, MGK, MP and FZ collected the whole data, treat the patients and performed the follow-up assessment while contributed to the final version of the paper. GND and ET wrote the final version of the paper. All authors have seen and approved the final draft of the paper.
